# Application of the humanized mouse model in research into SARS-CoV-2 infection (Review)

**DOI:** 10.3892/mi.2025.293

**Published:** 2025-12-29

**Authors:** Xiaoyue Feng, Yadong Wang, Yuan Li, Jinzhao Long, Fang Liu, Haiyan Yang

**Affiliations:** 1 Department of Epidemiology, School of Public Health, Zhengzhou University, Zhengzhou, Henan 450001, P.R. China; 2 Department of Toxicology, Henan Center for Disease Control and Prevention, Zhengzhou, Henan 450016, P.R. China

**Keywords:** COVID-19, severe acute respiratory syndrome coronavirus 2, humanized mice, pathogenesis, drug discovery, vaccine development

## Abstract

The coronavirus disease 2019 (COVID-19) pandemic triggered by the severe acute respiratory syndrome coronavirus 2 (SARS-CoV-2) has had a profound impact on global public health. The complexity of its pathogenic mechanisms and host interactions urgently requires high-fidelity animal models to support research. Humanized mouse models break the species barrier through gene editing and immune reconstitution technologies, providing a key tool to simulate human infection characteristics and pathological processes. A number of studies have reported the application of humanized mouse models in the fields of COVID-19 research, such as SARS-CoV-2 pathogenesis, anti-SARS-CoV-2 drug discovery and vaccine development, etc. The present review aimed to systematically document the latest advances in the application of humanized mouse models based on different construction strategies, such as receptor humanization, immune system humanization and composite humanization. These models have not only elucidated the pathogenicity differences and immune escape mechanisms of SARS-CoV-2 variants, but have also validated the efficacy of broad-spectrum anti-SARS-CoV-2 strategies, including angiotensin-converting enzyme 2-targeted therapies, antibody cocktail regimens and mucosal vaccines. Additionally, humanized mouse models have played a pivotal role in investigating the mechanisms underlying long COVID. By revealing the multi-system pathogenic mechanisms of pulmonary fibrosis, neurodegeneration and intestinal microbiota dysregulation, these models provide a theoretical foundation for the development of targeted intervention strategies.

## 1. Introduction

At the end of 2019, a novel coronavirus disease (COVID-19) caused by severe acute respiratory syndrome coronavirus 2 (SARS-CoV-2) spread rapidly around the globe, causing an unprecedented impact on the public health. SARS-CoV-2 belongs to the genus β-coronavirus, a single-stranded positive RNA virus with a genome length of ~30 kb (1). Among the structural proteins encoded by its genome, the spike protein consists of the S1 (S protein 1) and S2 (S protein 2) subunits, and binds to angiotensin-converting enzyme 2 (ACE2) on the surface of the host cells via a receptor-binding domain (RBD), mediating viral invasion and triggering infection. The process relies on the activation of S protein cleavage by host proteases, namely transmembrane serine protease 2 (TMPRSS2) (2). The highly transmissible nature of the virus allows for widespread dissemination through droplets, contact and aerosolization, resulting in progression from asymptomatic infection to severe pneumonia, acute respiratory distress syndrome and multi-organ failure with heterogeneous clinical presentations (3,4). Elderly patients with comorbid diseases have significantly higher rates of severe illness and mortality, highlighting the key role of host genetic background and viral interactions in the process of the disease (5). The long-term consequences of viral infections are gradually becoming apparent, with ~10-30% of recovered patients experiencing persistent fatigue, cognitive impairment and multi-system functional abnormalities; this phenomenon has been defined as long COVID (6).

**Figure 1:**
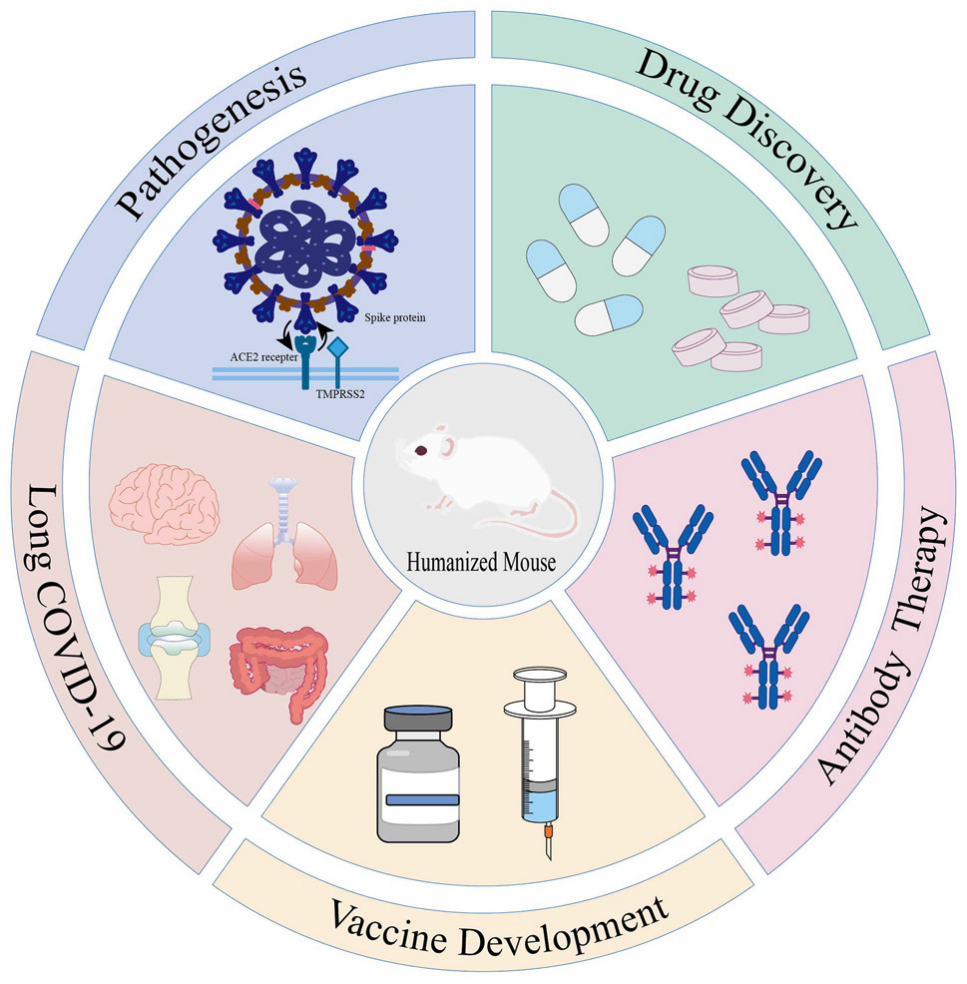
The application of the humanized mouse model in research into severe acute respiratory syndrome coronavirus 2 infection. COVID-19, coronavirus disease 2019.

In order to reveal the viral pathogenesis and develop intervention strategies, it has become imperative to establish animal models that can mimic human pathological characteristics. The mouse, as the most convenient laboratory mammal, is an ideal candidate due to its well-characterized genetic background and short reproductive cycle. While studies have identified alternative transmembrane proteins, such as CD147 and CD26 that may facilitate viral entry, ACE2 remains the primary gateway for SARS-CoV-2 to enter host cells (7). However, a natural species barrier exists: The mouse ACE2 (mACE2) receptor differs from human ACE2 (hACE2) at key amino acid residues, rendering mice naturally insensitive to SARS-CoV-2(8). This biological bottleneck has driven researchers to develop humanized mouse models through genetic engineering and immune reconstitution techniques. These models introduce human genes, cells, or tissues into immunodeficient mice to overcome species restrictions and replicate human infection characteristics. Numerous studies (9-13) have demonstrated the utility of humanized mouse models in COVID-19 research, including applications in viral pathogenesis, antiviral drug discovery and vaccine development. The present review aimed to systematically document the construction strategies of receptor humanization, immune system humanization and composite humanization models. We highlight their translational value in elucidating virus-host interactions, developing broad-spectrum antiviral therapies, optimizing antibody-based treatments and investigating the pathophysiology of long COVID (Fig. 1).

## 2. Strategies for the construction of humanized mouse models

**Figure 2:**
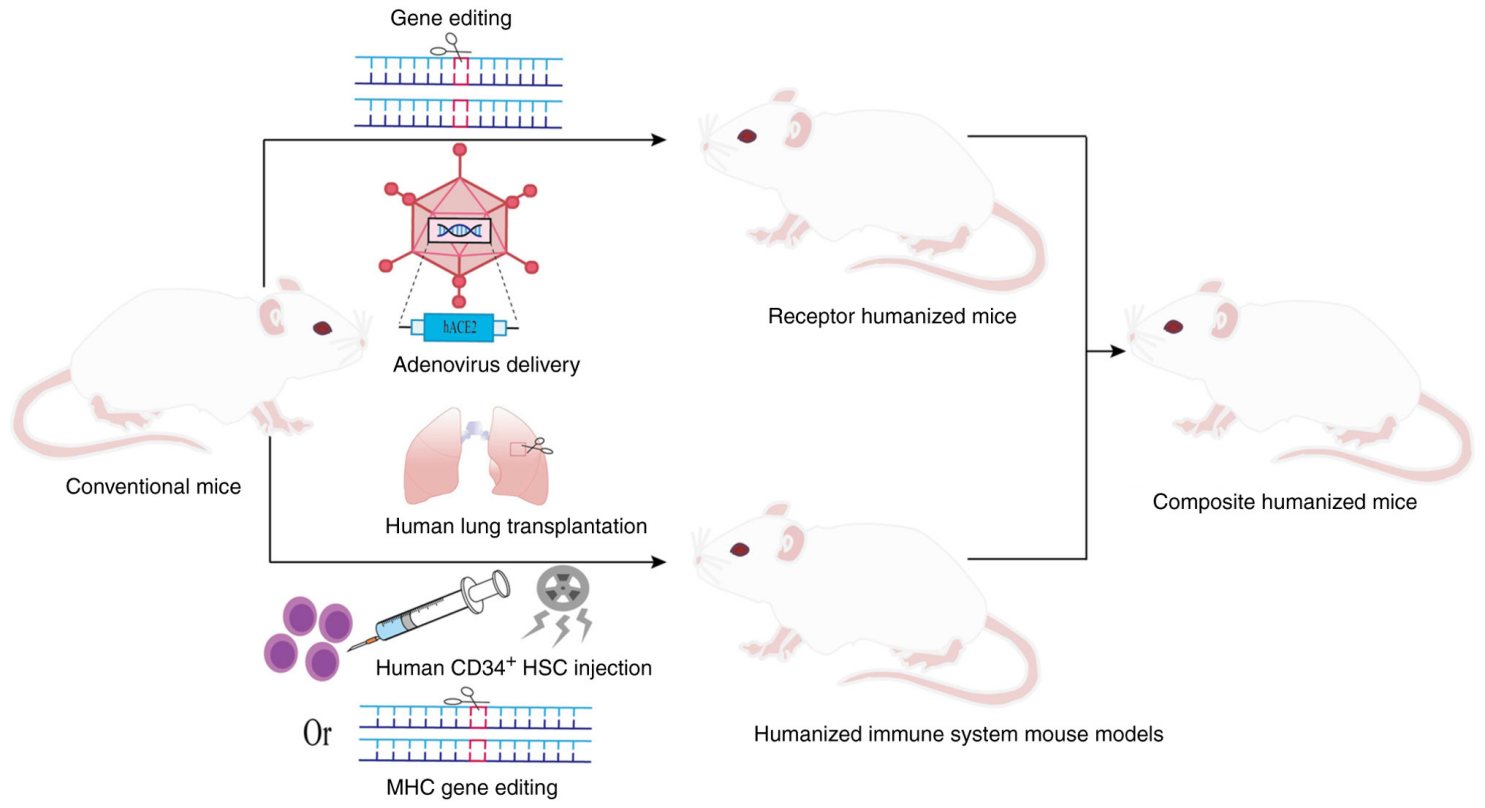
Strategies for constructing humanized mouse models. ACE2, angiotensin-converting enzyme 2; HSCs, hematopoietic stem cells; MHC, major histocompatibility complex.

To overcome the species barrier and simulate human SARS-CoV-2 infection, a variety of humanization strategies have been employed (Fig. 2), as described below.

### Receptor humanized mouse models

Receptor humanized mouse models were constructed to systematically mimic the functional properties of human receptors. K18 (cytokeratin 18)-hACE2 transgenic mice expressing hACE2 under the K18 promoter are susceptible to SARS-CoV-2(14). In the hACE2 mouse model created by Snouwaert *et al* (15) using homologous substitution, mouse promoter-driven ACE2 was highly expressed in airway club cells, whereas human promoter-driven ACE2 was expressed in alveolar type II cells. Ge *et al* (16) developed a fully humanized ACE2 mouse model that achieves tissue distribution and expression levels closer to those of human ACE2, thereby avoiding the expression abnormalities caused by heterologous promoters. Using clustered regularly interspaced short palindromic repeats (CRISPR)-Cas9 to introduce a single amino acid substitution (*H353K*) in the mouse ACE2 gene, the mACE2_H353K_ mouse model was successfully constructed (17,18). The application of complementary embryonic stem cell and tetraploid techniques has greatly accelerated model construction (19). hACE2-knock-in (KI) mice constructed by knocking hACE2 into mice via CRISPR-Cas technology have been used to study the mechanisms of pulmonary-intestinal dual infections and intestinal flora disruption and the pathology of tau protein in long COVID (20,21). Similarly, the generation of hACE2-KI-NOD/SCID gamma (NSG) mice by replacing mACE2 with hACE2 in NSG-immunodeficient mice significantly enhances their viral susceptibility (22). Furthermore, using hACE2 and/or hTMPRSS2 KI mice, researchers have found that Delta strains utilize membrane fusion for host entry, whereas Omicron strains employ endocytic pathways (23).

The use of conditional regulation systems enables the spatiotemporal control of receptor expression. Beyond hACE2-floxed (flanked by loxP sites) mice constructed by Cre recombinase-mediated knockout (24), an inducible bi-gene system was developed to tandemly connect hACE2 with green fluorescent protein downstream of the floxed STOP cassette, achieve spatiotemporal specific expression by tamoxifen-induced Ubi-Cre (ubiquitin promoter-driven Cre recombinase) (25). This research was extended to non-ACE2 receptors and synergistic mechanisms through the humanized dendritic cell (hDC)-specific intercellular adhesion molecule-3-grabbing non-integrin (SIGN) mouse model constructed by replacing mouse CD209a with human DC-SIGN (26), as well as through a humanized transferrin receptor (hTfR) mouse model (27) and a humanized model of the CD147 receptor (28). Among them, the severity of pulmonary fibrosis in the hCD147 mouse model exceeds that of the conventional hACE2 model. A summary of the general information on receptor-based construction of humanized mouse models is presented in Table I.

### Humanized immune system mouse models

The major histocompatibility complex (MHC) is known as the human leukocyte antigen (HLA) system in humans and is crucial for adaptive immunity. In the humanized mouse models of the immune system, a DRAGA mouse model was constructed by the post-irradiation infusion of HLA-matched umbilical cord blood-derived CD34^+^ hematopoietic stem cells (HSCs) and combined with transgenic technology to stably express HLA in the thymus (29,30). This design not only avoids graft-vs.-host disease but also promotes immunoglobulin class switching in human B cells, enabling long-term stable immune system reconstitution. Humanized HLA transgenic mice mimic human T-cell epitope presentation and post-vaccination immune responses, which avoid the severe pathological manifestations caused by the high susceptibility of traditional hACE2 transgenic mice (31,32). Furthermore, the humanized mouse model of the localized lung was constructed by transplanting human lung tissues into immunodeficient mice. In the SCID-hu model, transplanted human fetal lung tissues developed mature bronchioles, alveolar sacs, and vascular systems, and expressed the key receptor ACE2, providing target cells for SARS-CoV-2 infection (33). Similarly, the NSG-L model demonstrated efficient viral replication in grafts through the subcutaneous implantation of human lung tissue (34).

### Composite humanized mouse

The construction of composite humanized mouse models can more comprehensively and accurately simulate human infection and immune response to SARS-CoV-2 than a single humanized mouse. The MISTRG6 model supports the development of human myeloid and lymphoid lineage cells by replacing the corresponding mouse genes with human homologs. This model further achieves the dual humanization of the immune system and receptor expression by combining adeno-associated virus delivery of hACE2 to the lung (35-37). Similarly, the respiratory tract and immune system humanization in immunodeficient mice is achieved by the adenoviral delivery of the hACE2 gene to the lung of NIKO mice, combined with CD34^+^ HSCs transplantation (38). The HHD-DR1 model combines immune system and receptor humanization by knocking out mouse MHC molecules, introducing human MHC alleles, and driving hACE2 expression in the lung and brain (39). Additionally, the Alpha variant was found to be more infectious than the earlier virus 614D in the TKO-BLT-L mouse model constructed by transplanting bone marrow, liver, thymus and autologous lung tissue grafts (40). The HNFL mouse model constructed by combining human lung tissues with myeloid-enhanced immune system revealed the central role of myeloid immune cells in protecting lung tissues from SARS-CoV-2 infection (41). It is recommended that constructing single-, dual- and triple-gene composite models through precise knock-in of human ACE2, TMPRSS2 and Fcγ genes can meet the requirements for co-expression of multiple receptors (42).

In summary, the humanized mouse models constructed by various strategies lay an important foundation for the study of the pathogenic mechanism of SARS-CoV-2, the discovery of anti-SARS-CoV-2 drugs and the development of vaccines.

## 3. Application of the humanized mouse model in research into SARS-CoV-2 infection

### Research on the pathogenesis of SARS-CoV-2

The humanized mouse model has greatly enhanced the understanding of the pathogenesis of SARS-CoV-2 and provides a key basis for targeted therapy and for predicting the progression of the disease. Ye *et al* (43) demonstrated that, in hACE2 mice, that the virus targets non-neuronal olfactory epithelial cells, causing structural damage, immune cell infiltration and the downregulation of olfactory receptor genes, mirroring the transient anosmia of patients with COVID-19. A previous study using the K18-hACE2 model revealed that SARS-CoV-2 induces non-classical NLR family pyrin domain containing 3 (NLRP3) inflammasome activation via caspase-11, promoting the release of IL-1β/IL-18 and aggravating lung inflammation and mortality (9). In hACE2 mice, the RBD binding of the SARS-CoV-2 spike protein to ACE2 on mast cells triggers aggregation, degranulation, histamine and protease release, causing tracheobronchial and lung inflammation (44,45). Sefik *et al* (37) further demonstrated that, in MISTRG6-hACE2 mice, infected macrophages activated the NLRP3 inflammasome following viral uptake via CD16 and ACE2, driving chronic lung fibrosis. Additionally, neutrophils combat infection via phagocytosis, cytotoxic mediators and neutrophil extracellular traps (NETs). In K18-hACE2 mice, NETs trap SARS-CoV-2 particles; however, their excessive release damages tissue, highlighting the dual role of NETs (46). Notably, host factors play a key role in viral pathogenesis. Chuang *et al* (47) found that, in hACE2-KI mice, spike protein enhances viral susceptibility by activating ACE2 phosphorylation, inhibiting its degradation and promoting exosomal ACE2 propagation. The SARS-CoV-2 virulence factor, open reading frame (ORF)8 enhances pathogenicity by modulating host immune dynamics, including early interferon enhancement and late inflammatory suppression. Its deletion has been shown to reduce the mortality of K18-hACE2 mice by 40% (48). These models not only clarify the pathogenic mechanisms of COVID-19, but also provide a basis for immunomodulation strategies, such as inflammasome inhibition.

The interplay between the age, sex and genetic background of the host, and viral virulence significantly influences the disease course of COVID-19. For example, aged hACE2 mice infected with SARS-CoV-2 exhibit high mortality rates due to suppressed early inflammatory responses in lung endothelial cells. Additionally, the mortality rate in middle-aged male mice following high-dose infection (80%) far exceeds that in females (40%), reflecting the age and sex-related risk profiles observed in human cases of COVID-19 (49,50). Conversely, Haoyu *et al* (51) demonstrated that hACE2 mice with premature aging exhibit only mild pathology post-infection, indicating that progeria itself is not a direct risk factor for severe COVID-19. Furthermore, Snouwaert *et al* (15) identified tissue-specific and sex-specific differences in ACE2 expression in hACE2 mice. García-Ayllón *et al* (52) observed that the reduction in full-length ACE2 and the increase in truncated forms following SARS-CoV-2 infection in K18-hACE2 mice closely resembled the ACE2 dynamics during the acute phase of infection in humans. The expression levels and isoform dynamics of ACE2 provide further insight into the molecular basis of host susceptibility.

### Research on anti-SARS-CoV-2 drug discovery. Targeting of receptors to inhibit viral entry/replication

Lu *et al* (53) found that ACE2-specific antisense oligonucleotides downregulated the expression of respiratory ACE2 in K18-hACE2 mice. They inhibited SARS-CoV-2 and the its viral replication of its variants, reduced lung pathology and improved the survival rates of mice (53). ACE2 decoys are genetically engineered soluble ACE2 proteins that mimic natural ACE2 receptors, preventing viral entry by ‘tricking’ the virus into binding to them. Engineered ACE2 decoys, such as ACE2_2_.v2.4-IgG1, have been shown to broadly neutralize multiple SARS-CoV-2 variants. They have been shown to lead to significant reductions in lung injury and viral load in hACE2 mice (54-56). However, potential immunogenicity issues may limit their clinical application. Hwang *et al* (57) formed ACE2-incorporated nanodisc (NDAs), which mimic the host cell membrane structure to induce membrane fusion mediated by viral spike protein, leading to viral lysis and RNA release to directly kill the virus. A single intranasal NDA-Fc administration prevented lethal SARS-CoV-2 infection in K18-hACE2 transgenic mice (57). Similarly, Dong *et al* (58) found that coagulation factor FXa prolonged the survival of hACE2 mice by blocking the binding of SARS-CoV-2 spike protein to ACE2. Beyond ACE2, SARS-CoV-2 uses other co-factors for entry. Histamine receptor H1 binds viral spike protein independently of ACE2 and synergizes with ACE2 to enhance viral invasion. Yu *et al* (59) found that antihistamines could prevent SARS-CoV-2 infection in hACE2 mice by blocking this synergistic interaction, reducing viral entry and supporting the new use of antihistamine drugs.

Inhibitors targeting key enzymes of viral replication exhibit significant promise. The small-molecule compound 172 can target the 3-chymotrypsin-like protease variant of SARS-CoV-2 and inhibit its dimerization to block viral replication. Chan *et al* (60) found that compound 172 significantly reduced the viral load in K18-hACE2 mice and demonstrated broad-spectrum antiviral activity against various SARS-CoV-2 variants and human coronaviruses. Additionally, SARS-CoV-2 infection upregulates the host cathepsin L (CTSL), which facilitates viral entry by cleaving S protein. Zhao *et al* (10) observed that amantadine, a CTSL inhibitor, significantly reduced viral load and alleviated pathological lung damage in hACE2 mice, highlighting the potential of CTSL as a therapeutic target. Moreover, the SARS-CoV-2 spike protein induces metabolic reprogramming in hACE2 mouse hepatocytes. Mercado-Gómez *et al* (61) found that metformin reduced the risk of SARS-CoV-2 infection and attenuated metabolic disorders by modulating ACE2 expression and metabolic pathways, providing a novel therapeutic approach for patients with metabolic liver disease and COVID-19. Given the high mutation rate of SARS-CoV-2, developing drugs targeting host factors is essential to prevent resistance. Frasson *et al* (62) identified multiple SARS-CoV-2 variants co-dependent on host genes involved in oxidative stress and mitochondrial function-related pathways. The antioxidant *N*-acetylcysteine significantly reduced variant infection in hACE2 mice by inhibiting reactive oxygen species production required for early viral replication (62). Notably, Deshpande *et al* (63) found that SARS-CoV-2 infection may alter the pharmacokinetics of metabolic enzymes and transport proteins in hACE2 mice. This underscores the need for a systematic assessment of drug-host interactions and optimization of clinical drug safety.

*Modulation of the host immune-inflammatory response*. Immunomodulators can halt the vicious cycle of excessive inflammation. Lysine demethylase 1 (LSD1), involved in cell differentiation and inflammatory response, regulates the balance of pro-inflammatory and anti-inflammatory genes through epigenetic mechanisms. Mazzarella *et al* (64) demonstrated that the LSD1 inhibitor, DDP38003, specifically suppressed inflammation, preserved IFN-mediated antiviral activity in K18-hACE2 mice, and blocked viral release using the lysosomal acidification pathway; this enhanced IFN-independent antiviral mechanisms and balanced inflammation control, with antiviral effects (64). Another study demonstrated that the IL-1 receptor antagonist, anakinra, significantly reduced lung edema and fibrosis in K18-hACE2 mice by inhibiting NLRP3/IL-1β inflammatory signaling and restoring lung endothelial VE-calmodulin expression (65). Botella-Asunción *et al* (66) found that the novel anti-inflammatory drug, AG5, mitigated excessive inflammatory responses by reducing the release of IL-1β and IL-6, while preserving innate immune function in K18-hACE2 mice. The intranasal administration of immune adjuvants enhances non-specific innate immune responses early in viral replication and reduces morbidity and mortality. Weiss *et al* (67) reported that the intranasal immune adjuvant, N-dihydrogalactochitosan, reduced morbidity and mortality in SARS-CoV-2-infected K18-hACE2 mice by 75% through the activation of mucosal innate immunity. Similarly, it has been demonstrated that circulating galectin-9 (gal-9), with immunomodulatory properties, binds specifically to the host ACE2 receptor. Recombinant humanized gal-9 significantly alleviated acute-phase lethal infection in K18-hACE2 mice, offering a potential intervention strategy for COVID-19 therapy (68).

The study by Meier *et al* (69) observed that, in hACE2 mice, the pre-infection administration of losartan exacerbated inflammation, apoptosis and cognitive deficits, while post-infection administration attenuated these effects. Similarly, lisinopril upregulated ACE2 and increased viral load in hACE2 mice, while suppressing inflammation to reduce lung injury and thrombosis (70,71). These findings indicate that renin-angiotensin-aldosterone system modulators may either protect organ function through anti-inflammatory effects or exacerbate infection by promoting viral invasion. Their use should be carefully considered based on their dual effects on ACE2, with the timing of administration and type of drug being key determinants of clinical outcomes.

### Research on anti-SARS-CoV-2 antibodies. ACE2 competitive antibody

The interaction of RBD with host cell ACE2 receptors is critical for SARS-CoV-2 invasion. Clinical antibodies mostly neutralize the virus by blocking this interaction, and some of these also enhance the antiviral capacity through Fc-mediated effector functions, such as the clearance of infected cells and the activation of immune responses (72). For example, monoclonal antibody (mAb) ch2H2 and h11B11 both target the host ACE2 receptor to block viral binding. These antibodies exhibit broad-spectrum neutralization in the K18-hACE2 mouse model and are effective against various mutants, including the Omicron variant (11,73). Conversely, the 17T2 mAb targets a conserved region within the receptor-binding motif of the spike protein, maintaining pan-neutralizing activity against Omicron subvariants (BA.5, XBB) and demonstrating preventive and therapeutic efficacy in K18-hACE2 mice (74). Another notable mAb, NT-193, exhibits broad neutralizing activity against SARS-associated coronaviruses by targeting a conserved RBD site in the heavy chain and blocking ACE2 binding in the light chain (75). However, the efficacy of mAbs may be compromised by viral variation. Polyclonal antibodies (PAbs), which target multiple epitopes, may provide greater efficacy against mutant strains compared to mAbs. Vanhove *et al* (76) demonstrated that the polyclonal antibody, XAV-19, targeted multiple epitopes without inducing drug-resistant mutations. This antibody significantly reduced viral load in hACE2 mouse lung tissues, highlighting the potential advantages of PAbs in mitigating the impact of viral evolution (76).

Fu *et al* found that the mAbs, PR1077 and PR953, target the SARS-CoV-2 receptor-binding motif to directly block hACE2 binding. A single injection of PR1077 significantly reduced viral load in both prevention and treatment groups of Ad5 (adenoviral vector 5)-hACE2 transgenic mice (77). This suggests a potential strategy for developing monoclonal antibody cocktails to counteract viral resistance that may arise under the selective pressure of single-antibody therapies. For instance, the combination of REGN10933 and REGN10987, screened in humanized mice, formed a cocktail therapy to reduce the risk of SARS-CoV-2 escape (78). Similarly, Wang *et al* (79) demonstrated that combining JS026 and etesevimab significantly reduced viral load and alleviated lung pathology in hACE2 mice, exhibiting efficacy against the Alpha, Beta, Gamma and Delta variants.

Antibodies that simultaneously target multiple antigens with a single molecule provide advantages over traditional mAb cocktails. For example, the SARS-CoV-2 spike-targeting bispecific T-cell engager (S-BiTE) blocks viral entry and activates T-cell-mediated clearance of infected cells. Li *et al* (80) demonstrated that, in hACE2 mice, the viral load was significantly lower in the S-BiTE treatment group compared to the neutralizing antibody-only group, with comparable effects on the original strain and the Delta variant. Additionally, it was previously demonstrated that both bispecific humanized heavy chain antibodies and the IgG-VHH bispecific antibody, SYZJ001, exhibited protective efficacy in prophylactic and therapeutic experiments in hACE2 mice (81,82). Furthermore, Titong *et al* (83) found that intranasal prophylaxis with the tri-specific antibody, ABS-VIR-001, prevented infection and mortality in hACE2 mice, with a 50-fold reduction in viral load post-treatment. These findings highlight the potential of multi-specific antibodies in enhancing therapeutic efficacy and reducing the risk of viral escape.

*Non-ACE2 competitive antibody*. In addition to antibodies that block SARS-CoV-2 binding to ACE2 receptors, non-ACE2-competitive antibodies also play a role in neutralizing the virus. Yang *et al* (84) demonstrated that, in SARS-CoV-2-infected hACE2 mice, the antibody n3113 bound to the outer surface of the RBD and neutralized the virus by inhibiting spike protein-mediated membrane fusion. Similarly, another study demonstrated that the antibody, SP1-77, in humanized mice, did not prevent the virus from binding to ACE2, but instead blocked membrane fusion to achieve neutralization. This mechanism avoided a common pathway for viral escape (85). Furthermore, CD147 serves as a universal receptor for SARS-CoV-2 and its variants, which not only mediates viral entry into host, but also drives cytokine storms by modulating inflammatory signaling pathways. A previous study demonstrated that meplazumab, a humanized antibody targeting CD147, led to the broad-spectrum neutralization of SARS-CoV-2 and its variants in the hCD147 mouse model, providing a novel therapeutic strategy for COVID-19(86).

### Research on SARS-CoV-2 vaccine development

Humanized mice mimic the human immune response and are used in vaccine development to assess immunogenicity, safety and to explore new formulations and vaccination strategies. Turan *et al* (12) found that K18-hACE2 mice vaccinated with the inactivated OZG-38.61.3 vaccine had significantly lower mean viral loads in the high-dose group compared to unvaccinated infected controls, without observing significant toxicity, providing crucial evidence for clinical translation. Tai *et al* (87) demonstrated that an mRNA vaccine encapsulated in lipid nanoparticles induced a potent CD8^+^ T-cell response in humanized HLA transgenic mice. Freitag *et al* (88) investigated adenoviral vector 5 vaccines (Ad5-RBD and Ad5-S) and found that intranasal vaccination in humanized HLA mice elicited mucosal IgA/IgG-neutralizing antibodies and cytotoxic T-cell responses, providing effective protection against the Beta variant. Compared to intramuscular injection, intranasal administration avoids systemic viral vector dissemination, enhancing safety and presenting a viable alternative or supplement to existing vaccines. Gu *et al* (89) validated the Ad5 vector-based vaccine in the hACE2 mouse model, demonstrating no adverse effects on myocardial function even at high doses. This confirms its cardiac safety and provides an experimental basis for vaccinating high-risk cardiovascular patients (89). Additionally, García-Arriaza *et al* (90) demonstrated that the modified vaccinia virus Ankara (MVA) vector vaccine, MVA-S, exhibited potent immunogenicity and complete protective efficacy in hACE2 mice. A single dose prevented lethal infection, while two doses cleared the pulmonary virus, supporting its clinical translation (90). These findings highlight the potential of various vaccine platforms in combating SARS-CoV-2 and its variants.

While mRNA and viral vector vaccines have significantly advanced prevention strategies for COVID-19, their long-term safety, particularly in vulnerable populations such as children, pregnant women and immunocompromised individuals, and their cross-protective efficacy against emerging mutants, remain areas for improvement. Recombinant subunit vaccines provide a promising alternative due to their established safety and tolerability. For instance, Baiya-Vax-2, a recombinant plant-based SARS-CoV-2 RBD vaccine, induced high levels of neutralizing antibodies in K18-hACE2 mice, and two doses of the vaccine significantly lowered viral loads and prevented severe disease (91). The immunogenicity of the vaccine can be further enhanced by targeting strategies. Marlin *et al* (92) found that subunit vaccines targeting the viral antigen CD40-expressing antigen-presenting cells (αCD40.RBD) induced specific T-cell and B-cell responses in immune-system-humanized mice and established a persistent immune memory.

Vaccines have been effective in preventing COVID-19; however, concerns regarding vaccine-associated enhanced respiratory disease (VAERD) persist. Although the vaccine reduces viral load and mortality in hACE2 mice, it may trigger VAERD due to Th2/Th17 immune bias, manifested by pulmonary eosinophilic infiltration and IL-17-mediated systemic inflammation, which underscores the need for balanced immune responses and avoidance of the risk of Th2/Th17 pathology in vaccine development (93). T-cell epitopes are essential in immunity, with CD4^+^ T-cells regulating immune responses and CD8^+^ T-cells eliminating infected cells. Humanized MHC transgenic mouse models can generate specific cellular immune responses after inoculation with inactivated viruses, enabling rapid screening of T cell epitopes and accelerating vaccine design (94). Beyond conventional vaccine targets, regions of the virus not typically covered by existing vaccines are gaining attention as potential breakthroughs. The study by Weingarten-Gabbay *et al* (95) found that nonclassical ORF epitopes induced a more potent IFN-γ^+^ T-cell response associated with early viral protein expression and were more immunogenic than classical epitopes in immune-system-humanized mice. This suggests that non-classical epitopes may provide novel targets for vaccine design (95). Targeting highly conserved B-cell epitopes in spike protein or conserved T-cell epitopes in multiple coronaviruses induces potent neutralizing antibodies and cross-reactive T-cell responses in humanized mice. This approach markedly reduces viral load and attenuates lung inflammation (31,96). These findings highlight the central value of conserved epitopes in overcoming viral mutations and cross-species transmission, laying the groundwork for the development of a pan-coronavirus vaccine.

In summary, humanized mouse models not only provide an essential preclinical platform for refining current vaccines, but also represent a critical technology for addressing future coronavirus outbreaks.

### Research on long COVID-19 and complications

Although the COVID-19 pandemic has transitioned into a phase of normalized prevention and control, the potential risk of SARS-CoV-2-induced long COVID remains a key concern. Long COVID is defined as a multisystem syndrome with symptoms persisting for ≥12 weeks post-infection, characterized by pulmonary fibrosis, cognitive impairment, and multiorgan dysfunction (13). The heterogeneous clinical presentation of long COVID is linked to multifactorial pathogenic mechanisms, including persistent viral residues, autoimmune abnormalities, and chronic inflammatory cascades (97). Lung fibrosis is a hallmark of long COVID. Cui *et al* (98) simulated long COVID lung fibrosis in a humanized mouse model and found that chronic immune activation drove fibroblast differentiation and extracellular matrix deposition, leading to irreversible lung injury. Additionally, Heath *et al* (99) discovered that the SARS-CoV-2 spike protein disrupts the renin-angiotensin-aldosterone system (RAAS), exacerbating coagulation abnormalities and inhibiting fibrinolysis in hACE2-KI mice. This disruption leads to thromboembolic cerebrovascular complications and cognitive impairment (99). Similarly, the SARS-CoV-2 spike protein was found to exacerbate cerebrovascular oxidative stress and inflammation through the activation of RAAS-damaging pathways, resulting in vascular thinning and deteriorated cognitive function in diabetic hACE2 mice (100). These studies, leveraging humanized mouse models, have unveiled the pathological mechanisms of long COVID and provided a foundation for clinical interventions targeting immunomodulation and RAAS pathways.

Humanized mouse models have revealed that long-term residual SARS-CoV-2 RNA is associated with skeletal system abnormalities. Using K18-hACE2 mice, Haudenschild *et al* (101) found that the spike protein increased the risk of fractures by triggering osteoclast activation, bone loss and the thinning of the growth plate. This occurs either through direct binding to skeletal cell ACE2 receptors or indirectly through inflammatory hypoxia (101). Notably, SARS-CoV-2 can also infect the gastrointestinal tract and disrupt intestinal flora-host interactions. Research using the hACE2 mouse model has demonstrated that viral infection triggers intestinal barrier damage and flora disruption, characterized by a reduction in the amounts of beneficial bacteria and the proliferation of opportunistic pathogens. This leads to abnormally high flora diversity even in mild infections. Among these changes, the persistent reduction of the mucosal immune-critical bacterium *Akkermansia muciniphila* has been shown to be significantly associated with fatigue and gastrointestinal symptoms in long COVID (20,102). The study by Edwinson *et al* (103) using colony-humanized mice, further demonstrated that the gut flora reduces the risk of viral infections by inhibiting ACE2 expression. The regulation of ACE2 by healthy flora is stable, providing a theoretical basis for colony-targeted intervention strategies such as probiotic modulation (103). These findings indicate that the sequelae of COVID-19 not only involve the respiratory and cardiovascular systems, but that interactions between the skeletal, intestinal and immune systems may also lead to long-term health issues, underscoring the need for comprehensive management of the long-term effects of the virus.

## 4. Limitations

Although the present review systematically summarizes the key applications of humanized mouse models using in the research into SARS-CoV-2, certain limitations remain. Firstly, due to the uncertainty of ongoing virus evolution, the strategies for model construction (e.g., reliance on hACE2) and validation (e.g., a particular antibody or vaccine) are often based on ‘then known’ viral characteristics. The predictive value of the original highly specialized model may be reduced or even invalidated when a mutant strain with an altered invasion pathway emerges. Secondly, as regards the models themselves, even with the most advanced construction strategies, humanized mice struggle to fully replicate the complexity of the human immune system (38). On the one hand, these models are typically established on immunodeficient backgrounds, making it difficult to reproduce the complete immune network and interactions within the tissue microenvironment. On the other hand, the majority of models focus on reconstructing specific receptors or single-organ functions, failing to accurately reflect the systemic dynamic responses and multi-organ coordination mechanisms triggered by the virus (17,40). Simultaneously, fundamental differences exist between humans and mice in basic physiological structures, metabolic rates, and lifespan, resulting in significant shortcomings, particularly in simulating chronic pathological processes, such as ‘long COVID’. Additionally, the present review primarily relied on published literature and may not incorporate the latest preprints or ongoing research findings in a timely manner. In summary, while humanized mice provide a crucial platform for SARS-CoV-2 research, careful evaluation of their representativeness and applicability is essential when translating findings to clinical settings.

## 5. Conclusions

Humanized mouse models have been pivotal in uncovering SARS-CoV-2 pathogenicity and driving intervention strategies since the epidemic's onset. Through gene editing, receptor optimization and other technologies, researchers have constructed receptor humanized, immune humanized and composite humanized models to systematically simulate the infection characteristics and pathological process of COVID-19. Receptor humanization models have not only revealed the ACE2-dependent mechanism of viral invasion, but have also led to the discovery of the roles of novel co-receptors, such as CD147, TfR and DC-SIGN, which laid the foundation for multi-target drug design. In addition to breaking the species barrier by modifying viral receptors, the reconstruction of the human immune lineage can more comprehensively mimic the host immune response to COVID-19. Immune humanization models recapitulated the human-specific T/B cell response and the abnormal activation of myeloid lineage cells, and elucidated the roles of immune imbalance in long-term sequelae. By integrating human receptors and the immune system, the composite humanization model recapitulates the complex phenotypes of long-term viral retention, pulmonary fibrosis and neurodegeneration at the animal level, which provides important clues to analyze the mechanism of long COVID. These models validate the effectiveness of broad-spectrum antiviral strategies targeting ACE2, and they also accelerate the clinical translation of antibody cocktails, bispecific antibodies and mucosal vaccines.

## 6. Future prospects

Although significant progress has been made in the development of humanized mouse models, numerous challenges persist. Discrepancies in the spatial and temporal expression patterns of receptors compared to real human tissues can impact mechanistic accuracy. A novel live-virus-free mouse model has been developed, which obviates the need for viral adaptation or humanization. By administering GU-enriched ribooligonucleotides (mimicking the Delta/Omicron variant) via an oropharyngeal drip in combination with low-dose bleomycin-induced lung injury, this model successfully replicates key COVID-19 pathological features (104). Existing models predominantly focus on the acute infection phase, providing limited insight into long-term COVID-19 mechanisms, such as viral latency and reactivation or multi-organ interactions. Moreover, the low replication of the Omicron variant in some models reveals insufficient simulation of receptor-protease co-evolution. Further research is required to focus on developing modular rapid-response systems to keep pace with viral mutations.

The future development direction can break through from the following aspects, for model technology innovation, the integration of organoid and bioengineering models significantly improves the accuracy of pathology simulation. The humanized lung organoid-immunity chimeric model breaks through the bottleneck in the study of latent viral infections and trans-organ transmission by synchronously reconstructing the lung tissues and immune microenvironment (105). The two-dimensional air-liquid interface system constructed based on fetal lung bud-tip organoids accurately reproduced the specific infection of alveolar type II epithelium by SARS-CoV-2 and the interferon response mechanism (106). Bioengineered lung models dynamically simulate early COVID-19 infection characteristics by incorporating pathogen stimulation modules, with standardized construction systems supporting personalized medicine and large-scale drug development (107). Dynamic monitoring technologies combining *in vivo* imaging and single-cell sequencing enable the real-time analysis of infection processes. The ^68^Ga-NOTA-PEP4 PET imaging agent dynamically tracks changes in hACE2 expression (108), while radiolabeled pseudoviruses combined with SPECT/CT and PET imaging allow for the visualization of viral dynamics from invasion to clearance (109). For model optimization, the construction of a lung-intestinal-brain multi-tissue chimera model combined with an inducible viral latent system can systematically simulate the trans-organ damage mechanism of long COVID. Notably, the novel finding that the intestinal flora influences ACE2 expression through metabolic regulation suggests that the flora-immunity co-humanization model may become a critical breakthrough for revealing individualized susceptibility differences.

## Figures and Tables

**Table I:** Summary of the receptor-based construction of humanized mouse models.

SARS-CoV-2 strain	Mouse stain	Humanized manner	Mouse model	Main findings	(Refs.)
USA/WA1/2020	C57BL/6	hACE2 gene was inserted into the mouse and regulated by the K18 promoter	K18-hACE2 mice	Rapid humoral immune response in SARS-CoV-2-inoculated K18-hACE2 mice. Rapid systemic upregulation of pro-inflammatory cytokines and chemokines in SARS-CoV-2-inoculated K18-hACE2 mice.	(14)
SARS-CoV-2-MA10	C57BL/6 BALB/c	Replacement of specific regions of the mouse ACE2 locus using syntenic replacement	MP-ACE2 mice hACE2 mice	ACE2 expression in the lung and airways. ACE2 expression in lung club and AT2 cells.	(15)
USA/WA1/2020	C57BL/6	Introduction of key amino acid mutations in the mouse ACE2 gene by CRISPR/Cas9 technology. Insertion of hACE2 cDNA into the Rosa26 gene locus and conditionally expressed by the Cre-loxP system.	mACE2_H353K_ mice; Rosa26^hACE2^; CMV-Cre mice	Development of mice expressing mACE2-H353K. Isolation of mouse-adapted SARS-CoV-2. Mouse infection with SARS2_MA-H353K_.	(17,18)
Delta variant (B.1.617.2)	BALB/c	Replacement of the mACE2 gene with hACE2 using CRISPR/Cas9 technology	hACE2-KI mice	Viral responses were observed, and SARS-CoV-2 infection was confirmed in hACE2 mice. RNA sequencing of lung tissue unveiled unique transcriptional patterns, hinting at gut-lung interactions and enriched inflammatory pathways.	(20)
USA/WA1/2020 Delta(B.1.617.2); Omicron (B.1.1.529)	C57BL/6	Replacement of the mACE2 gene with hACE2 and insertion of a lox-stop-lox sequence in front using CRISPR/Cas9 technology	hACE2-KI mice	hACE2-KI mice inoculated with SARS-CoV-2 variants display distinct infectivity patterns. SARS-CoV-2 infection-induced tau pathology and ACE2 correlation in multiple brain regions of hACE2-KI mouse model.	(21)
USA/WA1/2020	NSG	Replacement of mACE2 gene with hACE2 by gene knock-in	hACE2-KI-NSG mice	S309-CAR-NK cell-treated hACE2-NSG mice have reduced SARS-CoV-2 viral loads in the lungs.	(22)
Delta variant (B.1.617.2); Omicron variant (B.1.1.529)	C57BL/6 BALB/c	Insertion of human genes into the corresponding positions of the mACE2 and mTMPRSS2 genes in mice using CRISPR-Cas9 technology	hACE2-KI mice hTMPRSS2-KI mice hACE2 and hTMPRSS2 double-KI mice	Disease course in SARS-CoV-2 d-and Omicron BA.1-infected single- and double-KI B6 and BALB/c mice. Infection pattern in SARS-CoV-2 d-and Omicron BA.1-infected single- and double-KI B6 and BALB/c mice.	(23)
USA-WA1 Omicron BA.1	C57BL/6	Insertion of hACE2 cDNA at the mouse ACE2 locus and add loxP sites. Insertion of loxP-stop-loxP-hACE2 at the Rosa26 locus.	hACE2-floxed mice LSL-hACE2 mice	hACE2^fl^ mice experience lethal infection following exposure to SARS-CoV-2. Lethal COVID-19 in hACE2^fl/y^ mice is associated with SARS-CoV-2 infection of the olfactory epithelium, olfactory bulb, and cerebrum.	(24)
Omicron (B.1.1.529)	C57BL/6	Replacement of the mouse DC-SIGN gene with the human DC-SIGN gene by CRISPR/Cas9 technology	hDC-SIGN mice	DC-SIGN gene humanization altered gut microbiota diversity in mice. The hDC-SIGN mice are susceptible to SARS-CoV-2 infection.	(26)
GD108 Omicron (B.1.1.529)	C57BL/6	Adenoviral vector (Ad5) delivery of the human gene TfR	hTfR mice hACE2 mice	TfR-mediated SARS-CoV-2 infection is ACE2 independent. Mice overexpressing hTfR are susceptible to SARS-CoV-2 infection.	(27)
Delta variant (B.1.617.2)	C57BL/6	Introduction of the human CD147 gene into the mouse genome	hCD147 mice	SARS-CoV-2 causes stronger fibrotic remodeling phenotypes in the hCD147 mouse model than in the hACE2 mouse model. The TGF-β-CD147 axis contributes to fibroblast activation in the lungs of a SARS-CoV-2-infected hCD147 mouse model.	(28)

SARS-CoV-2, severe acute respiratory syndrome coronavirus 2; hACE2, human angiotensin-converting enzyme 2; mACE2, mouse human angiotensin-converting enzyme 2; KI, knock-in; CRISPR/Cas9, clustered regularly interspaced short palindromic repeats associated protein 9; K18, keratin 18 promoter; AT2, alveolar type 2 cell; Cre-loxP, Cre recombinase/loxP system; hTMPRSS2, human transmembrane serine protease 2; TfR, transferrin receptor; TGF-β, transforming growth factor b; LSL, loxP-stop-loxP; hDC-SIGN, human dendritic cell-specific intercellular adhesion molecule-3 grabbing non-integrin; NSG, NOD/SCID/IL2Rγnull mice; Ad5, adenoviral vector 5; MP-ACE2,mouse ACE2 with partial humanization; CMV-Cre, cytomegalovirus promoter-driven Cre recombinase.

## Data Availability

Not applicable.
